# Advances in machine learning-based bacteria analysis for forensic identification: identity, ethnicity, and site of occurrence

**DOI:** 10.3389/fmicb.2023.1332857

**Published:** 2023-12-21

**Authors:** Geyao Xu, Xianzhuo Teng, Xing-Hua Gao, Li Zhang, Hongwei Yan, Rui-Qun Qi

**Affiliations:** ^1^Department of Dermatology, The First Hospital of China Medical University, Shenyang, China; ^2^Key Laboratory of Immunodermatology, Ministry of Education and NHC, National Joint Engineering Research Center for Theranostics of Immunological Skin Diseases, Shenyang, China; ^3^Department of Cardiology, Shengjing Hospital of China Medical University, Shenyang, China

**Keywords:** machine learning, deep leaning, artificial intelligence, forensic identification, microbiological

## Abstract

When faced with an unidentified body, identifying the victim can be challenging, particularly if physical characteristics are obscured or masked. In recent years, microbiological analysis in forensic science has emerged as a cutting-edge technology. It not only exhibits individual specificity, distinguishing different human biotraces from various sites of occurrence (e.g., gastrointestinal, oral, skin, respiratory, and genitourinary tracts), each hosting distinct bacterial species, but also offers insights into the accident’s location and the surrounding environment. The integration of machine learning with microbiomics provides a substantial improvement in classifying bacterial species compares to traditional sequencing techniques. This review discusses the use of machine learning algorithms such as RF, SVM, ANN, DNN, regression, and BN for the detection and identification of various bacteria, including *Bacillus anthracis, Acetobacter aceti, Staphylococcus aureus, and Streptococcus*, among others. Deep leaning techniques, such as Convolutional Neural Networks (CNN) models and derivatives, are also employed to predict the victim’s age, gender, lifestyle, and racial characteristics. It is anticipated that big data analytics and artificial intelligence will play a pivotal role in advancing forensic microbiology in the future.

## 1 Introduction

Microorganisms, encompassing fungi, bacteria and viruses, pervade both individuals and their surroundings, existing ubiquitously in nature. The exploration of the human microbiome, comprising microorganisms residing in, on, and around humans, has revolutionized our comprehension of the intricate interactions between these microorganisms and human health and disease (Johnson et al., 2016). The interplay of genetics and environment gives rise to a distinct microbiome for each individual. The skin microbiome is continuously shed and transferred from the host. Utilizing deposited microbes as supplementary evidence can aid in either including or excluding individuals in a criminal case (Sherier et al., 2022). Previous investigations have utilized microbial DNA markers, such as 16 s ribosomal RNA (rRNA), to evaluate the health of human hosts. However, this method is constrained to contaminated samples and hinges on the initial PCR amplification of labeled genes (Javan et al., 2016), limiting its applicability. The human microbiome presents a promising alternative for utilizing additional DNA sources in identifying or excluding individuals linked to erroneous evidence, thereby enhancing the efficiency of forensic DNA analysis in criminal investigations (Sherier et al., 2021).

Studies on the human microbiome have unveiled substantial variations in the composition and abundance of microbial communities across different body sites, varying states of host health, and among diverse racial groups. Notably, the skin flora is dominated by *Propionibacterium*, *Corynebacterium* and *Staphylococcus*, while the oral flora is characterized by the prevalence of *Lactobacillus* and *Haemophilus spp.*, and the gut flora is marked by the dominance of *Clostridium* and *Mycobacterium spp.* (Lei et al., 2022). For instance, previous investigations have highlighted significant differences in the microbiological composition of the hand microbiome alone, varying across countries and regions. In the hand microbiota, *Propionibacterium* and *Streptococcus* were prominent in Americans, but *Propionibacterium* was largely absent in the microbiota of Koreans and Japanese. Canadians, on the other hand, exhibited a hand microbiota mainly composed of *Bacillus*, *Streptococcus* and *Propionibacterium* (Cho and Eom, 2021).

**Figure 1:**
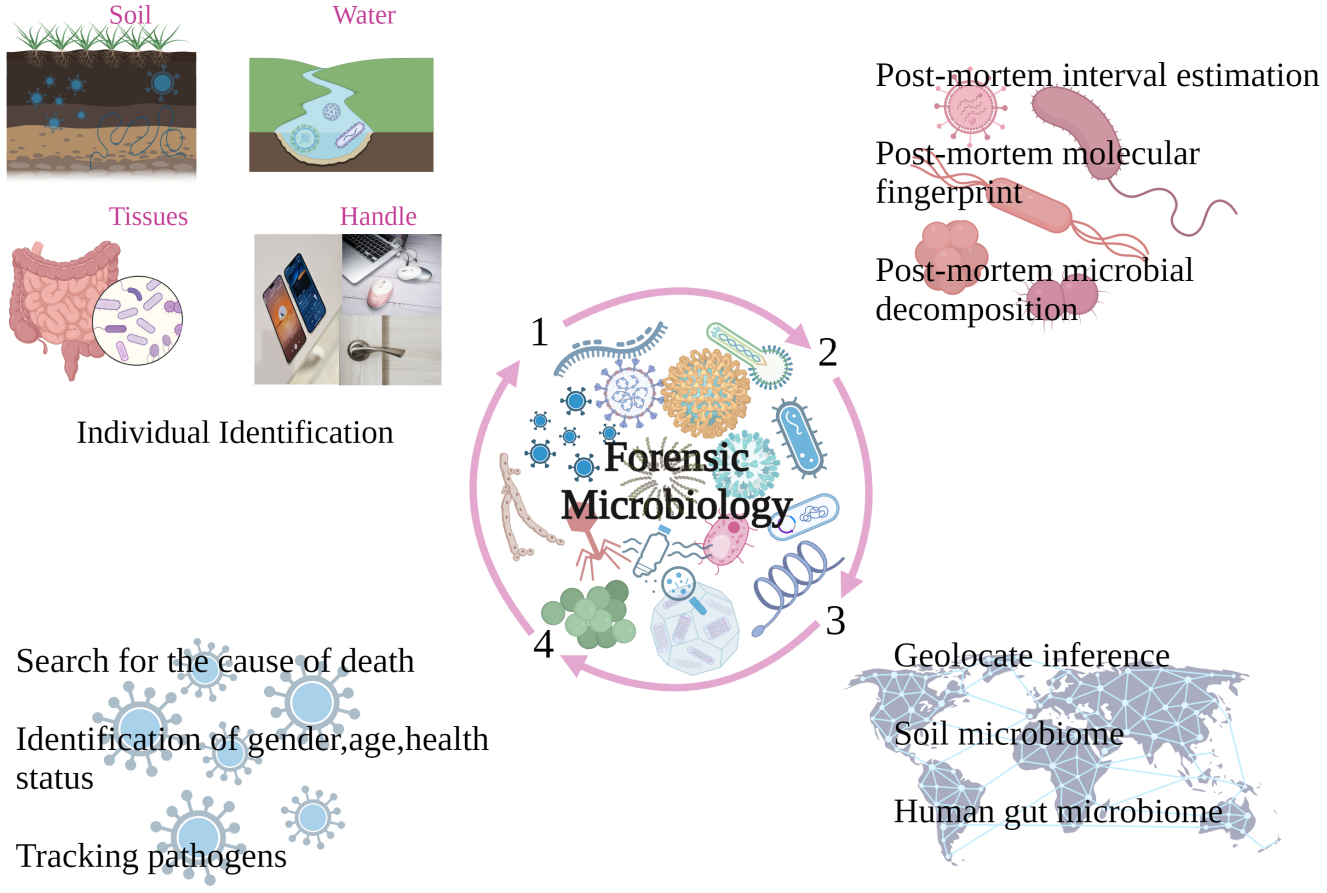
Forensic microbiological processes.

In recent years, the field of forensic microbiology has gained prominence in response to bioterrorist attacks. The primary objective of microbiological forensics is to employ diverse methods, including microbiology, molecular biology, immunology and analytical chemistry, to deduce potential mutant strains of microorganisms associated with bioterrorist attacks or natural disease outbreaks. It also aims to predict microbial origins, affinities or routes of transmission (refer to Figure 1). The changes in microorganisms following the death of the host remain poorly understood. In healthy individuals, the immune system typically prevents the colonization of internal organs and body fluids by microbes. However, after death, as the immune system and physical barriers break down, microorganisms proliferate throughout the body, starting in the gastrointestinal tract (Spagnolo et al., 2019). Consequently, the human post-mortem microbiome has been studied and identified as consisting of two components: microorganisms found in internal organs and body fluids after death, and microorganisms located on the surface of the remains postmortem, known as epinecrotic (Benbow et al., 2015). Research indicates that bacteria on the external surface of the body hold potential as markers for forensic identification. Skin bacteria, being highly resistant to environmental changes such as ultraviolet radiation, humidity and temperature, could serve as distinctive molecular “fingerprints” for humans (Javan et al., 2016). Furthermore, microbial decomposition processes play a crucial role in postmortem changes. While the succession of microbial communities is generally consistent across various soil types, the microbial community in the environment influences the decomposition processes of the cadaveric microbial community. As decomposing carcasses release a range of substances, including fatty tissues, volatile fatty acids, organic acids, organic nitrogen and specific anaerobic bacteria, into the soil (Wang et al., 2022), forensic identification based on microbial communities becomes feasible. Nevertheless, microbial communities alone are susceptible to external factors that limit their utility in forensic identification-a challenge that artificial intelligence (AI) may address by constructing effective assessment models.

**Figure 2:**
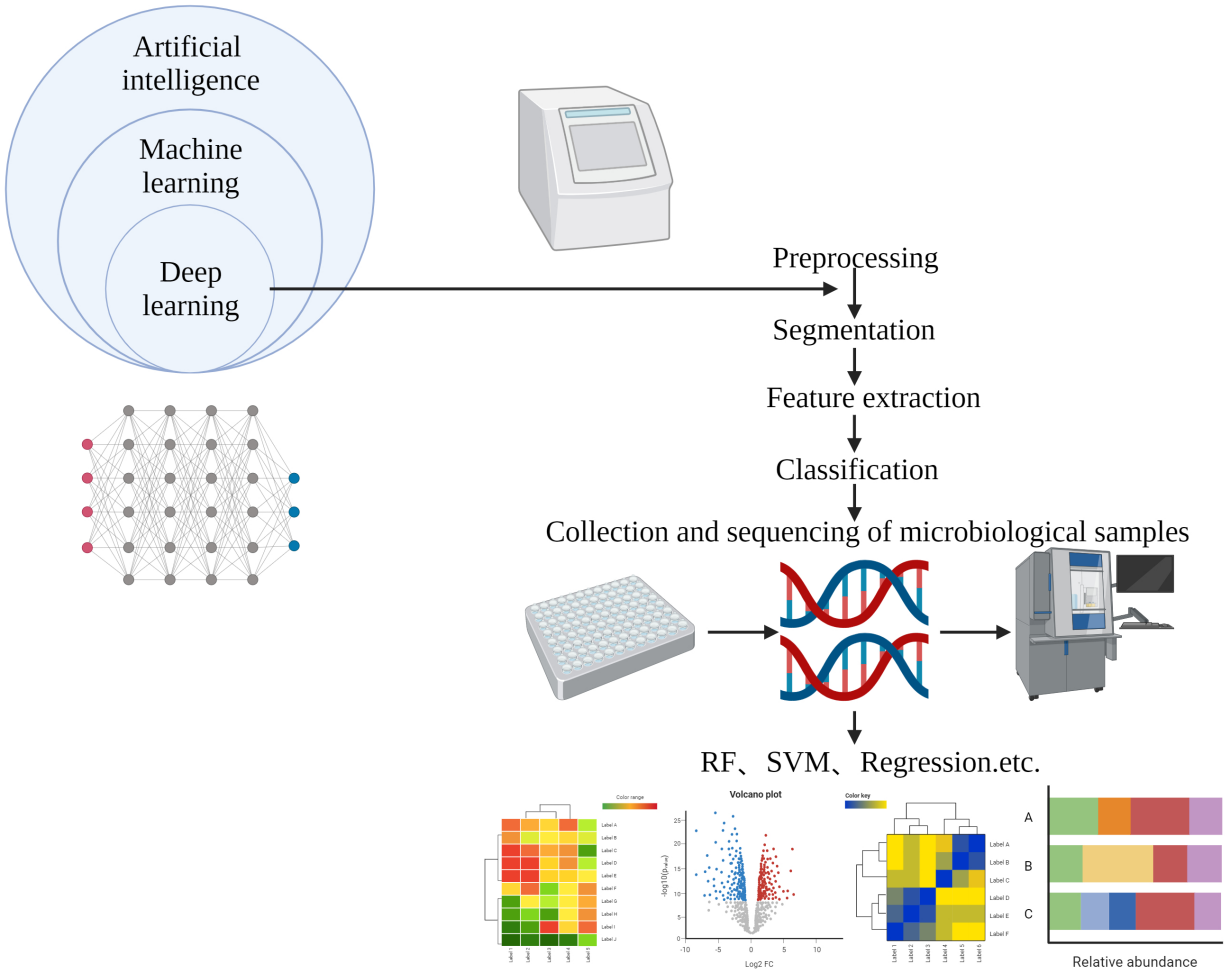
Artificial intelligence process.

Presently, machine learning (ML) stands as the primary AI technique employed in forensic research. ML, a subset of AI, devises algorithms to empower computers with the capability to learn without explicit programming (Handelman et al., 2018), as illustrated in Figure 2. Various ML methods, including Random Forest (RF), Support Vector Machines (SVM), Linear Regression, Logistic Regression (LR), among others, play a crucial role in this context. One notable subfield of ML is Deep Learning (DL), which encompasses Artificial Neural Networks (ANN), Multi-Layer Perceptron (MLP) networks, and Convolutional Neural Networks (CNN). The intersection of AI and microbiomics holds promise for a more profound understanding of the role played by microbial communities in cadavers. While conventional statistical methods are limited to determining the broad composition of microbial communities and their general successional changes, ML models facilitate quantitative analyses and accurate predictions (Yuan et al., 2023). This review integrates various AI models with forensic science to investigate the variability of microbial communities among different races and in diverse geographic environments (such as seawater, freshwater, soil, urban settings). The aim is to discern the individual identification, gender, age, and health of a victim or suspect. Moreover, suspect characterization can be conducted based on environmental microorganisms in the vicinity of the victim.

## 2 Application of machine learning in microbial forensic identification

Traditional autopsies come with numerous limitations, requiring substantial manpower and often leading to differences in expert opinions. AI emerges as a pivotal tool in various forensic science domains, addressing challenges in providing individual identity, scrutinizing body stains or marks, and identifying and collecting tissue or fluid samples. AI facilitates the comparison of field data with machine-generated data. By inputting an individual’s specific biometric pattern into the system, the machine swiftly matches it with pre-recorded biometric data to ascertain the individual’s identity efficiently (Wankhade et al., 2022).

Sherier et al. (2021) conducted a study where subjects’ non-dominant hands were sampled for skin microbes. They utilized the Hidskinplex multiple panels and SVM models to analyze markers and predict donor identity. The Hidskinplex is an innovative targeted sequencing panel comprising 286 skin core microbial markers, encompassing a taxonomy of specific microorganisms highly abundant on human skin (Schmedes et al., 2018). The results demonstrated that the classification accuracy of single nucleotide polymorphisms (SNPs) using the chosen method reached up to 94.77%. This conclusion was confirmed by the Schmedes et al. (2018). The research indicates that the use of a RF learning model can not only identify individuals by combining individual-specific bacteria (such as *Cutibacterium acnes*) with known genealogy data (Gu et al., 2022), but also predict different body parts. Schmedes et al. used the RF model to predict different body parts with an accuracy of 86%, while the Tackmann team’s prediction rate reached 89% (Yang et al., 2019), possibly because the Schmedes team only predicted three body parts: shirt collar, touched items, and feet, while the Tackmann team included a sample size of over 15,000. In 2022, Tackmann et al. (2018) collected and examined genetic data from Chinese, Korean, and Japanese populations based on publicly available database. They utilized the Softmax and RF methods to filter information, ensuring the reliability of classification results with an accuracy exceeding 90%, consistent with previous research findings. In summary, the use of various AI models allows for accurate identification of individual information and specific body parts. However, future efforts should involve a larger sample size for predictive statistical analysis. Subsequently, this elaboration delves into four key aspects of gender, age, health status and environmental cues—by amalgamting AI with microbiome research.

### 2.1 Gender identification

In recent years, the application of AI models in gender identification has proven to be valuable. A study found that the use of deep convolutional neural networks (DCNN) accurately predicted gender across diverse chest X-ray datasets from different regions (Li et al., 2022). Gao et al. (2018) introduced a novel method, MKDSIF-FCM (classification of skull datasets based on data mining and data analysis), which relies on unsupervised classification techniques for gender identification of Han skulls. The algorithm demonstrated a notable 98 and 93.02% gender prediction accuracy for females and males, respectively, suggesting its potential in forensic investigations. AI models not only demonstrate accurate predictive value in determining human gender but also yield similar conclusions in discerning the sex of dogs. Scarsella et al. (2020) employed a RF model to classify the microbial profiles of 340 healthy dogs based on gender factors. The results revealed that, compared to intact dogs, neutered males and spayed females formed distinct groups. These findings indicate a potential bidirectional interaction between microbial communities and host endocrine states. In the future, the integration of AI models based on microbial profiles holds greater promise in gender identification.

### 2.2 Age identification

Wang et al. (2021) developed a novel DL prediction method based on microbiome called MDeep. They applied this method to predict the age of 531 Americans by analyzing their gut microbiota. The results indicated that the MDeep model exhibited higher accuracy in age prediction compared to other learning models, as evidenced by a significantly improved overall R-squared value. Moreover, the MDeep model consistently demonstrated stability in the prediction (infant vs. child, child vs. adult) across sensitivity, specificity, accuracy, and precision metrics. Additionally, the model could assess the host’s age based on the maturity of the microbial community. Furthermore, MDeep proved effective in determining host age based on colony maturation. Subramanian et al. (2014) combined a sparse model with a 16 s rRNA dataset, resulting in the accurate prediction of the relative maturity and age score of the microbiota. Although promising, more research on microbiome-based AI for age prediction is still essential in the future to further provide a more robust theoretical foundation for forensic identification.

### 2.3 Health status identification

Adamker et al. (2018) employed three ML algorithms—LR, neural networks (NN), and SVM—to construct predictive models for anticipating *Shigella*, the most prevalent bacterium in Israel. The ML model demonstrated high accuracy in swiftly predicting the specific category of *Shigella*, aiding healthcare professionals in making real-time decisions about the need for hospitalization. Similarly, Njage et al. (2019) utilized the Logit Boost algorithm to predict the severity of outcomes following infection with *Shiga toxin-producing Escherichia coli* (*E. coli*), including symptoms such as diarrhea and hemolytic uremic syndrome, achieving an accuracy rate of up to 75%. If these models are integrated with whole-genome sequence data in the future, it could allow for the estimation of the health status of populations affected by the infection.

In addition to using microbiota to assess an individual’s health, it can also be employed to determine the cause of death in deceased individuals. The practice of scanning the entire body of a decreased individual using imaging methods, followed by computer software analysis to identify internal trauma and determine the actual cause of death, is referred to as virtual autopsy. Sullivan et al. (2017) have proposed the development of virtual biospecimen repositories enhanced with ML. The ML tool generates a series of non-invasive autopsy images utilizing X-rays, CT scans, or MRIs. Three images are then compared with the digital pathology data of corresponding biospecimens to comprehend the disease process. This approach aims to assess clinical diagnoses and treatments, representing an emerging trend in the field. More research on virtual autopsy is still needed in the future, but this method is poised to become a new trend.

### 2.4 Environmental identification

The compositional complexity and diversity of soils make them valuable for forensic identification. Soil pH, particularly in different geographical locations, plays a crucial role in shaping bacterial community (Gürtler et al., 2014). Ahmad et al. (2023) used various ML methods to classify soil rich in *Coxella* bacteria. They were able to identify correlations with elements such as clay, organic matter, trace elements, and slit, achieving a classification accuracy of up to 84.35%. Utilizing ANN assisted by Fourier-transform infrared spectroscopy, a ML approach was applied to differentiate *Bacillus cereus* group members in soil and foodborne outbreak samples. The study revealed that *psychrotolerant B. cereus* members in *Bacillus anthracis, B. cereus,* and *B. weihenstephanensis* dominated in soil samples from Germany, Malta and Switzerland (53–70%), with *Bacillus thuringiensis* in the minority (3–12%). In Danish soil samples, *B. weihenstephanensis* and *B. mycoides* were predominant (94%), with *Bacillus anthracis* accounted for only 6% (Bağcıoğlu et al., 2019). Consequently, examining soil microorganisms in the vicinity of a carcass may offer insights into predicting the location of death.

Microorganisms are also utilized for environmental identification in marine and freshwater settings. Ahmed et al. (2018) employed the SourceTracker program (an ML tool based on high-throughput sequencing data for microbial community tracking), studied the decay of sewage-associated bacterial communities in water. They found an increase in the abundance of cells from *Flavobacteriaceae* and *Spirochaetaceae* families in seawater. Similarly, in another study using the same program to detect bacteria in the middle and lower reaches of rivers in Russia, it was observed that during winter and summer, there was a significant increase in the abundance of *E. coli* and *Enterococci*; rod-shaped bacteria proliferated in sediments and other environmental habitats, showing host specificity. Moreover, *Flavobacterium*, *Sphingobacterium*, and *Serratia* were identified as the most common bacteria in freshwater ecosystems, with the first two being abundant in debris particles and algal plants. *β-Serratia* emerged as the predominant bacterium in organic aggregates and in streams with a high debris load (Dubinsky et al., 2016).

Furthermore, the use of microorganisms enables the identification of living environments. In the daily environments with which humans interact, approximately 360,000 micro-organisms are released per hour, presenting a valuable resource for forensic analysis. Furthermore, the human microbiome encompasses rare microbial taxa, forming a unique microbial spectrum. Lax et al. (2014) employed a Bayesian model to conduct a six-week monitoring of microbial community changes in seven American households. They discovered that the greatest differences in microbial species occurred in floor environments, while microbial populations on doorknobs were the most similar. Additionally, associations were identified in the distribution of microorganisms across various surfaces in homes. In addition, the discovery of 4,728 novel bacterial species over a span of three years in global urban public transportation systems has expanded the diversity of known urban microbiomes. This findings enhances the analysis of microbial interactions between humans and urban environments (Wu et al., 2022). Huang et al. (2020) employed LR and L2 normalization method to infer city affiliation in microbial samples, achieving an accuracy of up to 80%. Given the significant impact of urban microbial communities on human life, there is an ongoing need for future research focusing on the intricate interrelationships between human microbes and urban microbes.

## 3 Discussion

The progress of microbiology has been significantly propelled by the rapid advancements in high-throughput sequencing, bioinformatics, and AI technologies. From bacteria culture techniques in the 1990s to amplicon sequencing and metagenomics methods in recent years, and to AI models in the past two years, these advancements have greatly promoted the development of microbiology. AI can establish identity recognition, with machines inputting bodily parameters such as facial features, fingerprints, and retinal information. Machines record these parameters to establish individual identities. The recognition of individuals through biological data is referred to as biostatistics. The synergy of microbiomics and AI offers distinct advantages in terms of temporal stability, geographical variation in characteristics, and automatic prediction, contributing to the field of forensic identification. However, alongside their potential benefits, they also present numerous challenges. Uniform standards for evaluation and collection of data have not been universally established, and there is a need for large quantities of biometric data, despite challenges in infrastructure and resources availability. The diversity in AI methods poses challenges in terms of standardization and interoperability, and issues such as cross-contamination between samples and host DNA contamination need to be addressed. Looking forward, with the expansion of and the accumulation of data from microbial images, ML and even DL technologies are expected to wield even greater influence. The integration of AI and microbiomics holds promise in enhancing investigative opinions and conclusions with higher accuracy and timeliness, furnishing additional clues and a solid basis for criminal cases investigators. Continued advancements in both fields and the resolution of current challenges will likely contribute to the further refinement and application of these technologies in forensic science. In the future, machine learning models can facilitate the integration of various species of microorganisms, applying them practically through processes such as detection, data transmission, and analysis.

## Author contributions

GX: Investigation, Writing – original draft. XT: Writing – original draft. X-HG: Writing – review & editing. LZ: Writing – review & editing. HY: Writing – review & editing. R-QQ: Writing – review & editing, Conceptualization, Supervision.
